# Experimental and Modelling Study of the Effect of Adding Starch-Modified Natural Rubber Hybrid to the Vulcanization of Sorghum Fibers-Filled Natural Rubber

**DOI:** 10.3390/polym12123017

**Published:** 2020-12-17

**Authors:** Mochamad Chalid, Yuli Amalia Husnil, Santi Puspitasari, Adi Cifriadi

**Affiliations:** 1 Metallurgical and Material Engineering Department, Universitas Indonesia, Depok 16424, Indonesia; 2 Chemical Engineering Department, Institut Teknologi Indonesia, Banten 15320, Indonesia; yuli.husnil@iti.ac.id; 3 Research Center for Rubber Technology, Indonesian Rubber Research Institute, Bogor 16128, Indonesia; shanty_bptkbgr@yahoo.co.id (S.P.); cifriadi9748@gmail.com (A.C.)

**Keywords:** coupling agent, compatibility, rubber composites, kinetic study, vulcanization, natural fibers

## Abstract

Natural rubber-starch copolymer hybrid obtained from our laboratory was used as an additive for rubber compound. In this work, the effect of adding this hybrid material to vulcanization kinetics of sorghum fibers-filled natural rubber was studied. The rubber compounds were added with hybrid material at various loadings, i.e., zero to two phr and thus cured at three different temperatures, i.e., 130, 140, and 150 °C. The molecular behaviors due to the hybrid addition were investigated by Fourier-Transform Infrared (FTIR) spectroscopy. The rheological phenomena of the rubber compounds were studied by performing torque analysis in moving die rheometer. The obtained data were utilized to develop the thermodynamic modeling. The compatibility of sorghum fibers-natural rubber blends in the presence of starch-modified natural rubber were characterized using Field Emission Scanning Electron Microscope (FE-SEM). FTIR results show noticeable changes in the peak intensity of particular functional groups from rubber and natural fiber as evidence of molecular interaction enhancements between rubber and natural fibers caused by incorporating the starch-modified natural rubber coupling agent to rubber-natural fiber blends. The curing time for these blends was reduced with lower required activation energy. SEM images show no visible gaps in morphology between natural rubber and the filler indicating that the addition of hybrid material to the blends also improves the compatibility between the fibers and the rubber matrix.

## 1. Introduction

Natural rubbers (NR) are important materials due to having suitable properties for many applications such as high-performance tires, automotive parts, and adhesives, to name a few. Considering its shortcomings, such as low aging resistance, thermal instability, and poor mechanical properties, NR products have been developed through some approaches, for instance, by forming composites with other materials [1]. Silica, clay, carbon nanotube, and graphene are some of the synthetic materials that have been utilized as reinforcing agents for NR and were reported to be able to improve the mechanical properties of the rubber [2]. The environmental concerns are the driving force to find alternative materials that are environmentally benign.

Natural fiber (NF) is a potential material for a reinforcing agent for polymers like NR due to its excellent properties and renewability. Sorghum, Kenaf, and Ijuk fibers are some of the fibers that have been utilized as reinforcing agents for other types of polymers [3,4,5,6]. Some authors also studied the effect of incorporating NF into an NR matrix to the properties of the composites [7,8,9]. These materials have exceptional properties such as being lightweight, low density, high toughness, good mechanical and thermal properties, being environmentally friendly, and higher resistance to fracture [10]. Among these, sorghum fiber has a superiority of high cellulose content leading to excellent mechanical properties [11]. However, utilization of NF as the reinforcement for NR has limitations due to its low compatibility (a low interfacial interaction) to an NR matrix. This phenomenon leads to poor dispersion and distribution of NF in an NR matrix, which can lead to unsatisfactory mechanical properties [12].

One of the methods to improve the compatibility between NR and NF is by adding a coupling agent to the composite that could act as the bridge that connects the two materials [13]. This agent is synthesized by chemically modifying the main structure of NR to create a new NR structure that consists of polar and nonpolar parts. In previous works, modifying the chemical structure of NR was performed through grafting a synthetic polar compound such as maleic anhydride [14], methyl methacrylate [15], acrylonitrile [16], and styrene [17] onto the backbone of the rubber [18]. In this study, we used a coupling agent that was synthesized through grafting biomass, i.e., starch to NR backbone producing hybrid material [19].

Starch was preferred as the grafting material to NR, considering its renewability and good acoustic properties [20]. Grafting can be performed through living-free radical method, e.g., atomic transfer radical polymerization [21]; radiation, e.g., peroxidation technique or plasma radiation [22]. The starch-grafted natural rubber used in this research was synthesized in our laboratory using glow discharge electrolyte plasma (GDEP), a method to initiate polymerization without using any chemical agent, catalyst, and organic solvent [23].

Aside from enhancing compatibility between filler and matrix, the coupling agent may also affect the kinetic behavior during rubber vulcanization [17,24,25,26]. Previous research indicates that the presence of filler in NR may increase the efficiency of the curing reaction of NR by lowering the activation energy [25]. Therefore, the kinetic behavior of the NR/NF composites that were added with NR-g-St was also studied to learn the effect of incorporating the coupling agent to the composites and how it will affect the activation energy of the curing reaction.

This study was dedicated to investigating the effect of adding starch-grafted natural rubber (NR-g-St) to the NR/NF composites on the compatibility between the NR matrix and the fiber as the filler. The experiments for this research were performed by adding NR-g-St to NR/NF blend at various loading, i.e., zero, one, and two phr. first, we conducted careful analysis on Fourier-Transform Infrared (FTIR) spectra of the resulting NR composites and observed the shifting of peaks of certain functional groups to understand the effect of incorporating this third component from a molecular interaction perspective. Based on our observation on the FTIR spectra, the mechanisms of the molecular interaction were proposed. Second, the compatibility between NR and NF was observed morphologically by using a Field Emission Scanning Electron Microscope (FE-SEM). Lastly, the resulted compounds were tested using a moving die rheometer (MDR) at three variations of temperatures to obtain the rheological measurement. The data were then regressed and fitted using Claxton–Liska and Deng–Isayev approach to analyze the kinetics parameters of the natural rubber curing [24].

## 2. Experiments

### 2.1. Materials

The natural rubber type SIR 20 with Plasticity Retention Index and Rapid Plasticity, 61.8 and 34, respectively, was provided by the Indonesian Rubber Research Institute (IRRI), Bogor, Indonesia. All additives to support the vulcanization process such as zinc oxide powder (ZnO), stearic acid, CBS (N-Cyclohexyl-2-benzothiazole sulfenamide), and sulfur were supplied by Multi Citra Chemindo Nusa, Ltd., Indonesia. Sorghum fibers based on the stem part were provided by SEAMEO BIOTROP in Bogor, Indonesia. The fibers were pretreated to obtain average aspect ratio of 25.62 ± 33.68. The starch-grafted natural rubber (NR-g-St) was synthesized from our laboratory based on the procedure in [19]. NaOH solids used in graft copolymerization of NR were obtained from Merck.

### 2.2. Methods

#### 2.2.1. Graft Copolymerization

Grafting of starch onto natural rubber was performed through method of glow discharge electrolyte plasma (GDEP) [17]. Starch powders were added into 5%-wt of natural rubber in water emulsion (latex), until weight ratio of latex to starch was 5:1. Then, NaOH solids were added to the mixture until concentration of 0.04 M. The resulted mixture was then stirred for 5 min and then placed in GDEP reactor.

The GDEP reactor had a volume of 1 L and was maintained at 60 °C with cooling jacket, and 6-mm stainless steel (SS-316) and 0.5 mm tungsten (wolfram) was used as anode and cathode, respectively. The electrodes were immersed in the liquid mixture, separated at a distance 20 mm. Then, 658.3 volts were then charged to the electrodes to initiate the graft copolymerization. After 10 min, a solid product as NR-g-St was separated by filtering, followed by purification through washing using chloroform. Finally, the hybrid product was dried in a vacuum oven at 50 °C for 4 h. The degree of grafting of the resulted NR-g-St was 68%.

#### 2.2.2. Composite Production

In this study, all compounds of sorghum fibers and natural rubber mixture were prepared at 100 and 20 phr in composition, respectively. Referring to ACS 1 standard compound formula, the composition of ZnO, stearic acid, CBS, and sulfur in the rubber compound formula were prepared as 6, 0.5, 0.7, and 2.5 phr. While the composition of NR-g-St was varied as 1, 2, and 3 phr. NR that was not added with NF and NR-g-St is referred as ‘Blank’ throughout this paper. The detail composition of samples prepared in this work and its assigned names are presented in Table 1.

The NR compounds were produced using rubber compounding method by referring to ASTM D 3182. The procedure was started with mastication of natural rubber through rolling the rubber on the two rolled open mill of Berstorff (capacity of 1 kg/milling) at 60 °C for 3 min, followed with addition of the others such as ZnO and stearic acid, respectively. After mixing for 2 min, sorghum fibers and NR-g-St addition were added respectively, followed by adding CBS and sulfur after 2 min. The mixture of rubber compounds was homogenized by blending and re-milling for 3 min. Finally, the homogenous mixtures of rubber compounds were cured in an oven to obtain rubber composites; or in a system of rheometer to investigate the rheological behavior of the rubber compounds during curing (vulcanization) process.

#### 2.2.3. Fourier Transform Infrared (FTIR) Analysis

The composite products were characterized using FTIR Perkin Elmer 90325 (PerkinElmer Inc., Waltham, MA, USA) to study functional groups of molecules in the composite products and their interactions, as the results from previous processes such as curing. Furthermore, the FTIR spectra will be used to study the effect of adding NR-g-St in various loadings into the natural rubber compounds.

#### 2.2.4. Field Emission—Scanning Electron Microscope (FE-SEM) Observation

Effect of NR-g-St to the compatibility between NR and NF in the composites was investigated by characterizing morphology of the composite products. The observation of the morphology was carried out by using FE-SEM FEI Inspect F50 (FEI Company, Hillsboro, OR, USA).

#### 2.2.5. Rheometer Test

Thirty grams of NR composite samples with and without coupling agent were used for rheological measurement. The measurement was conducted by using a Moving Die Rheometer (MDR) Alpha 2000R (MonTech USA, Columbia City, IN, USA) at various temperatures (130, 140, and 150 °C). Furthermore, the rheological measurement obtains a rheometer curves indicating scorch time (t_s2_), optimum curing time (t_90_), maximum torque (M_H_), and minimum torque (M_L_). These parameters were used for qualitative analysis of molecular interactions that occurred during curing reaction and for calculating the kinetic parameters of the reaction.

#### 2.2.6. Crosslink Density

NR composites were shaped into a C-size dumbbell and tested using ASTM D412 to obtain stress-strain data. LF-Plus Universal Testing Machine with serial number LF1207-Lloyd Instruments (AMETEK, Inc., Berwyn, IL, USA) and software NEXYGEN Material Testing (AMETEK, Inc., Berwyn, IL, USA) were utilized for this measurement. The crosslink density of cured NR composites was measured by using the Mooney–Rivlin method where the stress-strain data were inserted into the following Equation (1) [27].

(1) $$\frac{\sigma }{2\left(\lambda -\lambda^{-2}\right)}=C_{1}+C_{2}\lambda^{-1}$$

where σ and λ denotes the stress (N/mm^2^) and strain (%), respectively. By plotting the left side of Equation (1) vs. $\lambda^{-1}$ the value of $C_{1}$ was obtained as the intercept of the graph. The elastic constant $C_{1}$ is related to crosslink density ($M_{C}$) through the mathematical expression shown in Equation (2) below [27].

(2) $$2C_{1}=\rho RT/M_{C}$$

## 3. Results and Discussions

### 3.1. Molecular Interaction

**Figure 1:**
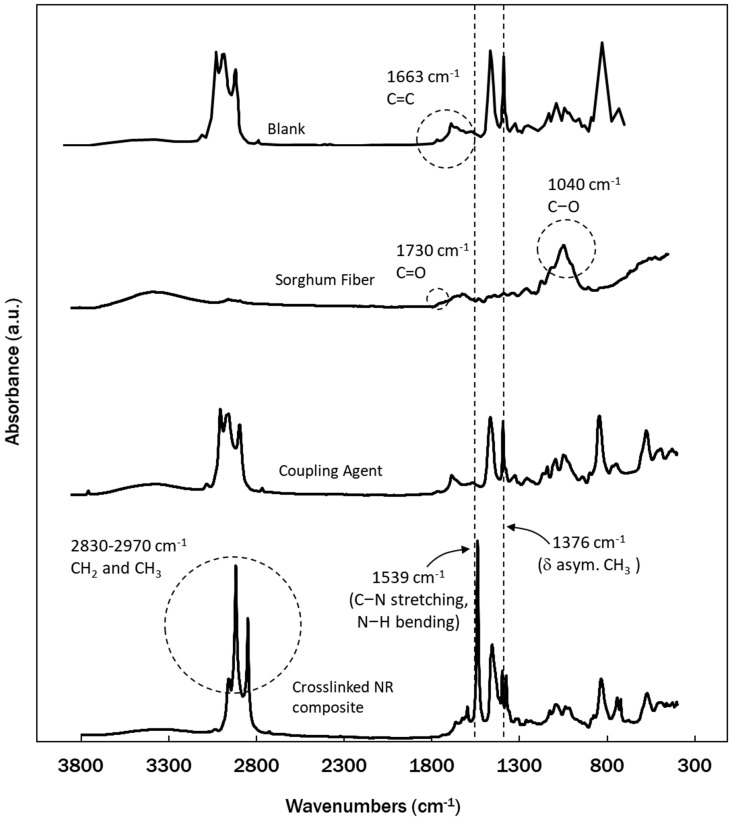
Fourier Transform Infrared (FTIR) spectra of natural rubber (NR), sorghum fibers, NR-g-St, and composites synthesized from those materials.

FTIR spectra of functional groups from NR, sorghum fibers, NR-g-St, and the composite obtained from those materials are presented in Figure 1. ‘Coupling Agent’ and ‘Crosslinked NR Composites’ in the figure refer to NR-g-St graft copolymer and NR/NF/NR-g-St composite, respectively. The FTIR spectra of NR and NR-g-St showed a peak at 1663 cm^−1^, which is a typical peak for carbon double bond found in cis-1,4 polyisoprene of natural rubber. As for the spectrum of sorghum fibers, peaks could be found at 1730 and 1040 cm^−1^, which are specific for ketone/aldehyde C=O stretch and C–O bond of amorphous cellulose, respectively. The typical peaks were also reported in previous research [28,29,30].

As a combination of chemical structures from starch (St) and natural rubber (NR), the FTIR spectrum of NR-g-St graft copolymer showed peaks that belong to functional groups found in NR as well as traces of peaks that refer to cellulose. However, the FTIR spectrum of Crosslinked NR Composites was slightly different. The absorbance values of some peaks such as the one for CH_2_ and CH_3_ groups were higher in ‘Crosslinked NR Composites’ compared to the same peaks found in ‘Blank’ and ‘Coupling Agent’. Also, the peaks were slightly shifting to a lower wavenumber. These changes in peak intensity and position indicated that there may be molecular interactions between functional groups in NR and NF that occurred during curing reaction, which were possibly induced by the presence of NR-g-St in the compounds.

As shown in Figure 1, the peaks at wavenumber range of 2830–2970 cm^−1^, unique for –CH_2_ and –CH_3_ bonds, were more pronounced in the spectrum for ‘Crosslinked NR Composite’. On the other hand, the peak at 1376 cm^−1^ showed less intensity compared to the spectrum of ‘Blank’. These changes in intensity were possibly due to the intermolecular interaction that occurred during the process.

There was also a noticeable peak change at 1539 cm^−1^ of crosslinked NR composite, which is in wavenumber range for amide II band (C–N stretching and N–H bending) [31]. These groups belong to the protein molecules in NR molecules that might have underwent a Maillard reaction with carbonyl groups in monosaccharides that make up sorghum fibers [32]. The reaction promoted protein crosslinking and thus increased the concentration of amide II in the composite [33].

**Figure 2:**
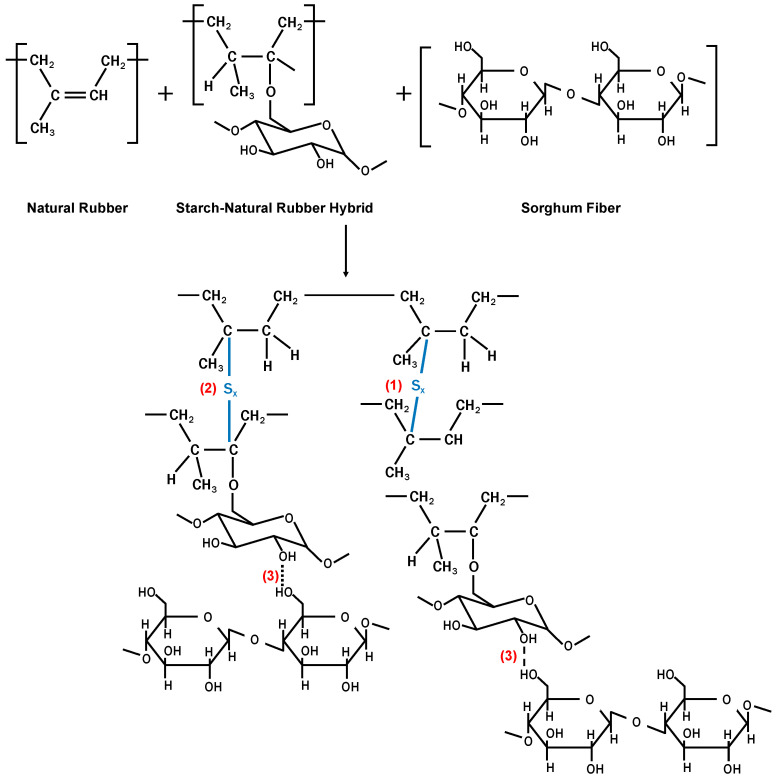
Proposed crosslinking bonds and intermolecular bonding in natural (sorghum) fibers-filled in natural rubber composite, added NR-g-St coupling agent.

**Figure 3:**
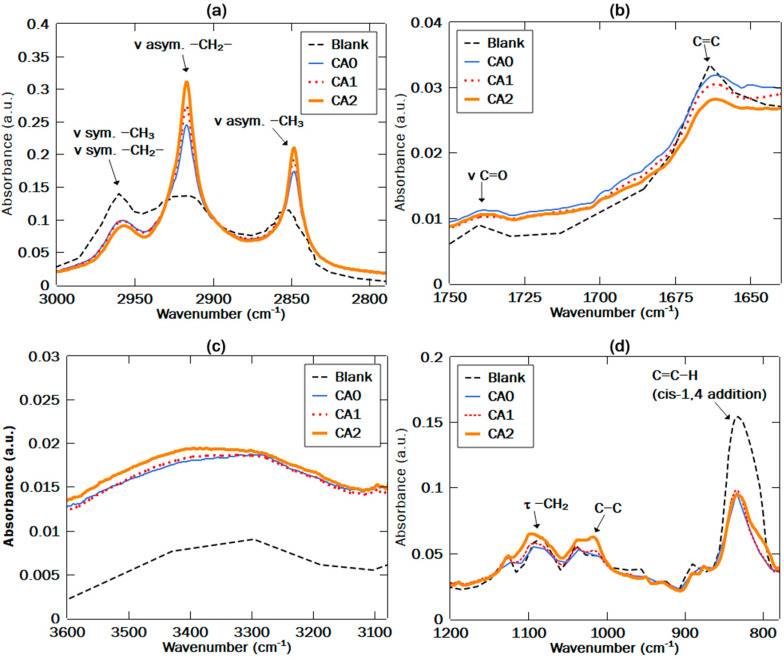
FTIR spectra in split wavenumber range (**a**–**d**) for natural (sorghum) fibers-filled in natural rubber composite, added NR-g-St coupling agent, and the Blank.

Referring to our previous studies [19,20] and FTIR spectra of the composite in Figure 1, there were some possible mechanisms of molecular interaction, which formed intermolecular bonds between NR matrix and NF fillers, as shown in Figure 2. The intermolecular bonds were built from crosslinking between NR backbones (1) and NR molecules with NR parts in NR-g-St graft copolymer (2). Figure 2 also illustrates interactions between NR matrix and NF (sorghum fiber) filler through a hydrogen bonding (3) between NF and the starch part in NR-g-St graft copolymer that was crosslinked to NR backbone. These proposed interactions due to the addition of NR-g-St to NR/NF composites will be further studied by careful analysis on FTIR spectra of the composites as shown in Figure 3.

Figure 3 shows FTIR spectra for vulcanized NR/NF/NR-g-St composites at various compositions of NR-g-St using ‘Blank’ as the reference. The FTIR spectrum for Blank in Figure 3a displayed three shoulder peaks at a wavelength interval from 2830 to 2970 cm^−1^, indicating functional group vibrations of NR backbones, such as ν asym. –CH_3_ at 2970 cm^−1^, ν asym. –CH_2_ at 2920 cm^−1^, and ν sym. –CH_2_ at 2850 cm^−1^, as also reported in the literature [31]. Absorbance intensities of the ν asym. –CH_2_ and ν asym. –CH_3_ peaks for CA0 to CA2 were more pronounced and higher than ‘Blank’. It may have been caused by the presence of CH_2_ on cellulose, hemicellulose, and lignin chains of sorghum fiber. Besides, starch backbone that contains CH_2_ group may have contributed to the enhancement of the absorbance intensity. Intensity of the peaks was proportional to the composition of the coupling agent. Figure 3b shows a peak at 1740 cm^−1^ corresponding to stretch vibration of the C=O in non-conjugated ketones, carbonyls, and in ester groups that may belong to the remaining lipids, neutral glycolipids, or phospholipids in NR. The absorbance intensity for this peak in the four composites were noticeably higher than in Blank. This may be caused by lignin and hemicellulose content in sorghum fibers.

Figure 3b also shows peaks at 1663 and 1010 cm^−1^ assigned to vibration of C=C and C–C bond, respectively. This finding was also reported by a previous study [31]. Compared to the FTIR spectrum of Blank, the absorbance intensities for C=C and C–C bond were decreasing and increasing, respectively, for CA0. This may have been due to the addition of sorghum fibers. A similar trend could also be found in CA1 and CA2. In this case, the change in the intensities of both functional groups was possibly due to the addition of the fibers and NR-g-St. These tendencies may have had a correlation to crosslinking bonds formation, as described in Figure 2. In detail, CA2 had the lowest absorbance intensity for C=C bond, whereas for C–C bond the absorbance intensity was highest. This was confirmed by the increasing absorbance intensity of symmetrical and asymmetrical –CH_2_ group in Figure 3a, which was possibly also related to this alteration of C=C to C–C bond for crosslinking bonds formation. The replacement of C=C bond into C–C bond consequently induced a proton transfer that changed C–H bond into –CH_2_ bonds. Furthermore, this premise was confirmed by the FTIR spectra in Figure 3d, showing a significant decreasing of the absorbance intensity of C=C–H peaks due to addition of NR-g-St coupling agent.

As discussed in Figure 2, molecular interactions between NR matrix and NF filler were bridged by NR-g-St coupling agent through crosslinking and hydrogen bonding. In Figure 3c, the hydrogen bonding was indicated by peak at 3308 cm^−1^, as confirmed in the literature [30]. The figure indicates a significant gap of absorbance intensity between the Blank and the composites; and an increasing of absorbance intensity from CA0 to CA2.

**Figure 4:**
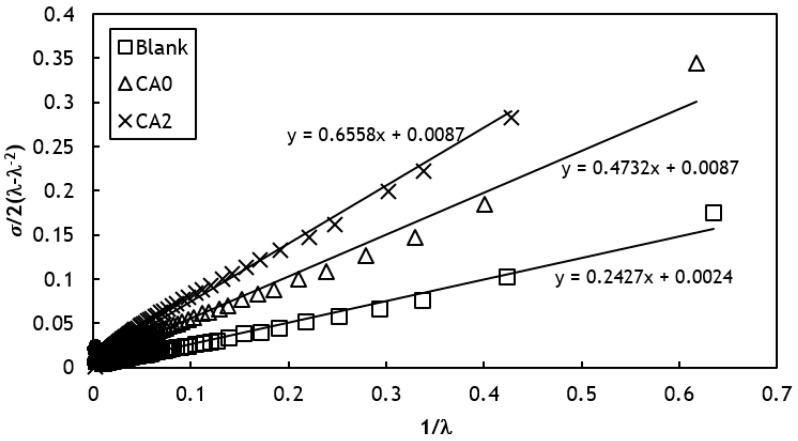
Linear regression of Mooney–Rivlin plots to obtain intercept (C1) as parameter for calculating crosslink density for Blank, CA0, and CA2.

The effect of adding NR-g-St to the molecular interactions in the composite can be studied by measuring the degree of crosslinking of the composite. The graphical result from stress and strain test data that were inserted into Equation (1) is presented in Figure 4.

Based on the Mooney–Rivlin method, the degree of crosslinking on the cured systems is represented by the intercept of the tangent line, denoted with C1. This value was then utilized to solve Equation (2) to obtain crosslinking density for NR composites. The crosslinking density for Blank, CA0, and CA2 were 1.93 × 10^−6^, 6.99 × 10^−6^_,_ and 6.99 × 10^−6^ mol/m^3^, respectively. The crosslinking density values confirm the effect of incorporating NR-g-St to the composite on its crosslinking behaviors. Furthermore, this behavior can be used as a parameter to investigate the effect of adding NR-g-St coupling agent to the properties of the NR composites such as compatibility between the matrix and the filler, as discussed in the next part.

### 3.2. Compatibility Study

**Figure 5:**
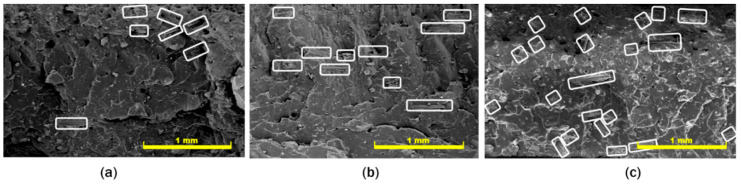
Field Emission-Scanning Electron Microscope (FE-SEM) images of fractural cross-section on natural fiber (NF) filled NR composites that were added with NR-g-St at compositions of (**a**) 0 phr, (**b**) 1 phr, and (**c**) 2 phr. All images are at 100× magnification.

Study of the compatibility between fibers and NR matrix was performed by using the FE-SEM to observe morphological behaviors on fractural cross-section of the composites, as shown in Figure 5. The composite without adding the coupling agent of NR-g-St, in Figure 5a showed an agglomeration of the natural fiber fillers and some voids, which may have been caused by the fibers in the natural rubber being pulled-out during mixing process. Furthermore, Figure 5b,c depict that the fibers in composites with 1 and 2 phr of NR-g-St were incorporated in the matrix with better distribution and dispersion compared to NR/NF composite without the coupling agent. These findings further support our analysis that NR-g-St acts as the bridge that connects NR and NF molecules. The coupling agent improved the interfacial interaction between the two materials as can be seen from the well distributed and dispersed NF filler in NR matrix.

**Figure 6:**
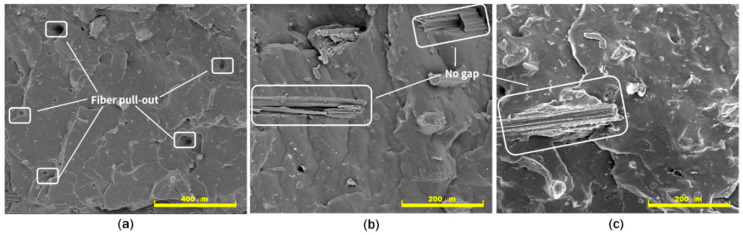
FE-SEM images of fractural cross-section on NR/NF composite added NR-g-St with (**a**) 0 phr at 100× magnification, (**b**) 1 phr, and (**c**) 2 phr, both at 500× magnification.

Further observation is presented in Figure 6a, which shows holes in the NR matrix. These holes indicated low wettability between NR and NF, which consequently resulted in poor interfacial interaction between the two materials. The fibers were pulled-out from the matrix during mixing process, promoting fiber agglomeration and finally creating limited dispersion and a low distribution of the fibers in the NR matrix. Figure 6b,c show morphology of NR composites that were added with 1 and 2 phr (CA1 and CA2) NR-g-St, respectively. The figures imply the fibers penetration into the NR matrix in a relatively better wettability, which was showed by no noticeable gaps among surfaces of the fiber and the matrix.

Referring to the discussion in Figure 1 and Figure 2, molecular interactions between NR, NF, and NR-g-St in the composite occurred through the formation of hydrogen bonding among polar parts of the filler and the coupling agent; and crosslink bonding among non-polar parts of the matrix and the coupling agent during vulcanization. The coupling agent implicitly has a function to bridge between the filler and the matrix, which led to enhance the compatibility between NF filler and NR matrix [34]. Good compatibility between fibers and NR matrix may consequently affect the rheological behavior of NR composite compounds, as discussed in the subsequent parts.

### 3.3. Rheological Behaviors

**Figure 7:**
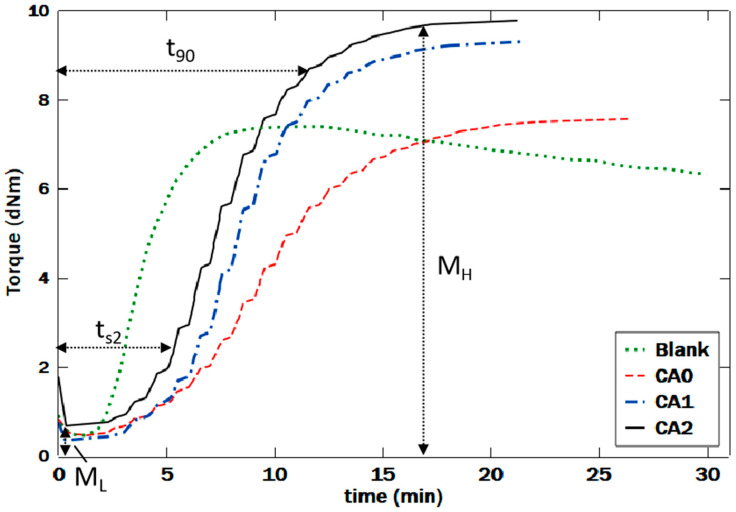
Rheological profile of the natural rubber composite compounds during measuring the rheometer in 150 °C.

The rheological behavior of the NR composites was studied by observing the rheology curves of the samples, as shown in Figure 7. These curves, overall, visualize the change in the stiffness feature of NR, which was represented by how much torque required for preserving the shear stress on the rubber, as function of time. As can be seen from all curves in the figure, in the first few minutes, the torque underwent a decreasing value, implying that the viscosity of the rubber compound was reducing with temperature. This shows that at this period, the compounds were still in Newtonian region [35]. This condition leads to the lowest torque (M_L_), the minimum stiffness or viscosity of the rubber composite compound before the crosslinking reaction. The values of M_L_ for Blank, CA0, CA1, and CA2 were 0.47, 0.46, 0.35, and 0.67 dNm, respectively. The lowest torque for CA2, which was highest compared to the others, may be caused by the presence of the novel coupling agent NR-g-St in NR/NF composites.

The crosslink between NR backbone with the coupling agent and the hydrogen bonding between the coupling agent and NF may have enhanced the wettability of the NF filler by NR matrix. These interactions lowered the molecular mobility of the matrix, hence increasing its viscosity, as also reported by previous studies [36]. After this point, the torque continuously increased until it reached highest torque, which indicated as the maximum stiffness of the rubber composite compound after crosslinking reaction. The maximum torques (M_H_) values are 7.37, 7.57, 9.3, and 9.78 dNm for Blank, CA0, CA1, and CA2, respectively. These values of M_H_ correspond to the crosslinking network structures of the natural rubber composite, i.e., the density and the length of sulfur-based crosslinking bridges [37], which was achieved after curing. A higher value of M_H_ for CA2 possibly contributed to the fact that it had the largest amount of NR-g-St in the composite. Since the coupling agent had both nonpolar and polar parts [20], it enhanced the crosslink between NR and NF molecules in CA2, thus decreasing the mobility of the macromolecular chain. It produced harder and stiffer rubber composite [38], indicated by the higher value of M_H_. Moreover, this mobility inhibition may be caused by the formation of the hydrogen bonding among the polar parts originating from NF and NR-g-St. Both illustrations are in line with discussion regarding the proposed molecular interactions (Figure 1), which are supported FTIR spectra in Figure 2.

The effect of adding NR-g-St to NR/NF compounds on crosslinking behavior of the composites during curing reaction can also be observed by analyzing the time parameter shown in the rheology curves. The curves show that the samples have different times to reach 90% of the maximum torque (t_90_), viz. 6.07, 15.8, 12.78, and 12.1 min as t_90_ for Blank, CA0, CA1, and CA2, respectively. Blank had the lowest t_90_ compared to the others, which may be attributed to the lack of filler in the sample. The crosslinking process between NR molecules in Blank was easier compared to the other samples that are more complex. Therefore, it took less time to reach 90% of M_H_ for Blank. The incompatibility between NR and NF may be responsible to the higher value of t_90_ of CA0. The curing reaction of this sample required more time because the presence of NF to some extent inhibited the crosslinking between NR molecules. As the composition of NR-g-St increases in NR/NF composites, the time needed to reach 90% of M_H_ reduces. This was possibly due to the act of bridging provided by NR-g-St, which resulted in more effective crosslinking between certain molecules in the composites with mechanisms as discussed in Section 3.1.

**Figure 8:**
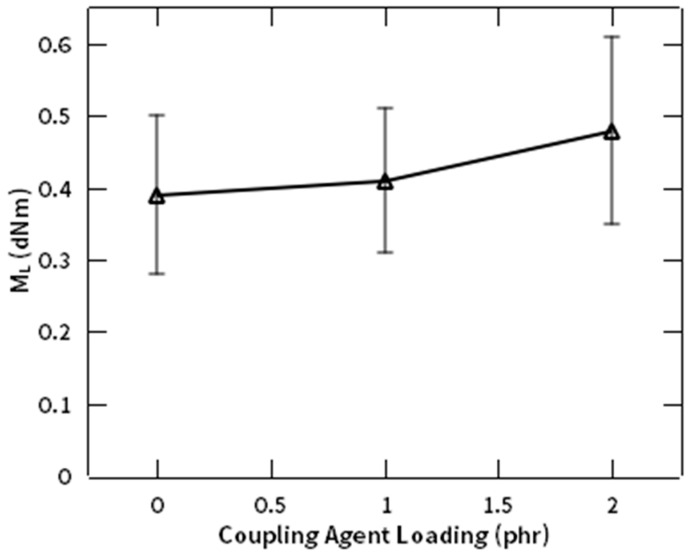
M_L_ profile of NR compound at different composition of NR-g-St.

Figure 8 illustrates in more detail the effect of NR-g-St loading on the previously mentioned rheological parameters of NR compounds during crosslinking. This figure implies that in rheological measurement at 130 °C, the trend line of M_L_ is proportional to the composition of NR-g-St in the compound. As there were more NR-g-St in the composites, the molecular interaction between NR matrix and NF filler were more reinforced, hence enhancing the compatibility of the filler and the matrix, as discussed in Figure 2.

**Figure 9:**
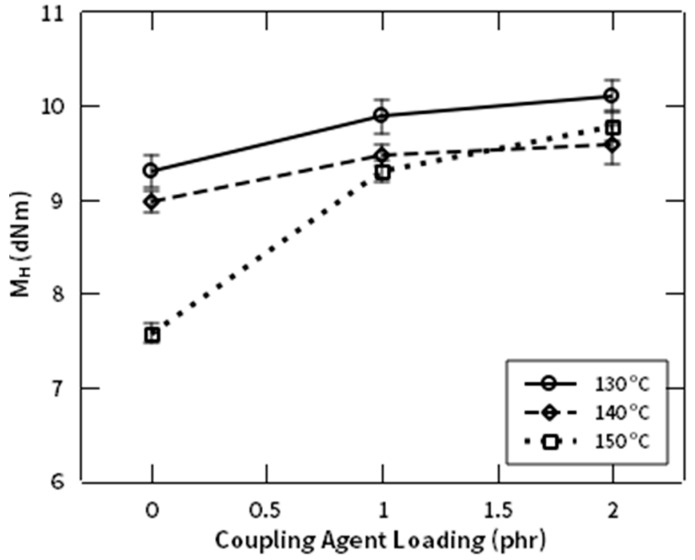
M_H_ profile of NR compound at different composition of NR-g-St.

Figure 9 shows the effect of NR-g-St composition to the crosslinking density achieved after curing reaction, indicated by parameter of M_H_ at 130, 140, and 150 °C. The figures show that crosslinking density is proportional to NR-g-St composition in all temperatures. This finding implies that the presence of NR-g-St in the rubber composites may lead to longer crosslinking networks (see discussions that are referring to Figure 1 and Figure 2). Increasing of NR-g-St composition in the composite compound therefore resulted in higher M_H_. This tendency is in line as reported by [36].

**Figure 10:**
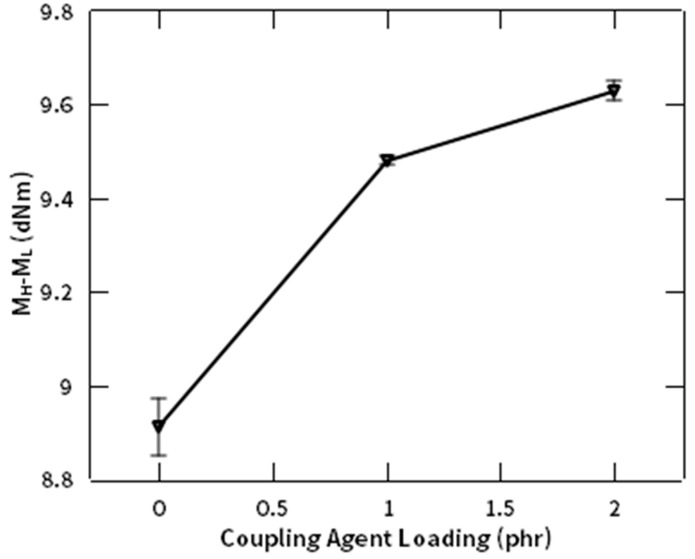
M_H_-M_L_ profile of NR compound at different composition of NR-g-St.

Furthermore, the above analysis needs to be confirmed by calculating the difference between maximum and minimum torque (M_H_-M_L_), as a measure of the crosslinking present in the system [25]. Figure 10 represents effect of NR-g-St composition in the composite, to value of the M_H_-M_L_ at curing temperature of 130 °C. The figure shows a proportional correlation between both parameters. The M_H_-M_L_ data from this study is generally in agreement with the said hypothesis. These results have a similar tendency with the previous study, which reported that the presence of filler in NR matrix will create bigger interlayer spacing where more NR molecules will be confined in it, thus promoting crosslinking reactions [39].

**Figure 11:**
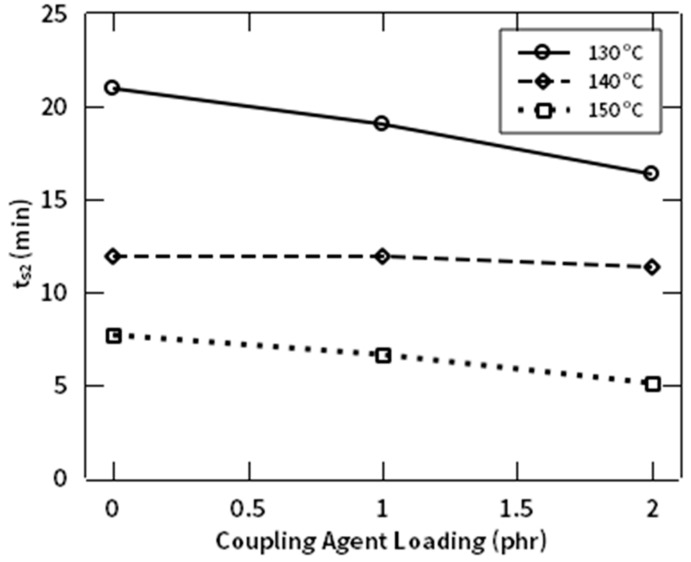
ts_2_ profile of NR compound at different composition of NR-g-St.

**Figure 12:**
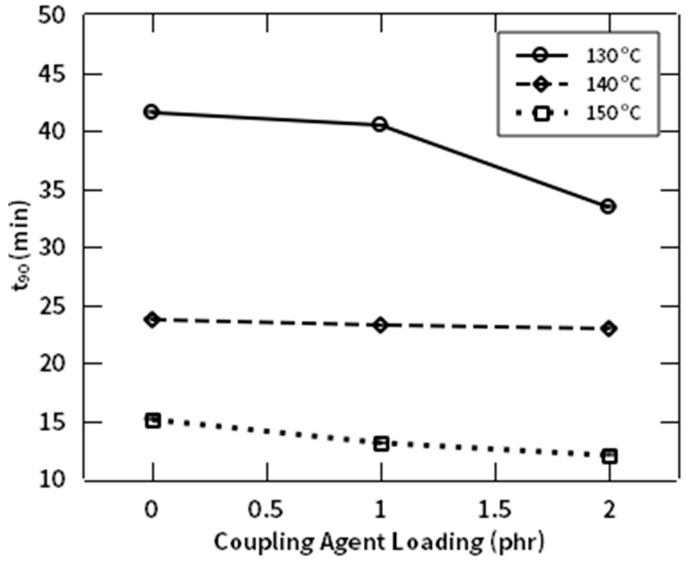
t_90_ profile of NR compound at different composition of NR-g-St.

The rheological behavior of NR/NF composites with and without addition of NR-g-St coupling agent were studied by observing the required time to achieve two torque units above M_L_, as the scorch time (ts_2_) and optimum crosslinking, as the t_90_, which are shown in Figure 11 and Figure 12, respectively. Figure 11 shows that increasing the composition of NR-g-St in the compound, at the same curing temperature, shifted the scorch time (ts_2_) to a shorter time, thus reducing the duration for accumulating the heat to initiate vulcanization of the composites. In other words, as the amount of the hybrid coupling agent increases, the required time and energy for initiating curing reaction decreases. It means that NR-g-St addition to the rubber compound accelerates the formation of crosslink precursors resulted from reaction between activated polysulfides with the unsaturated sites in NR backbone [40]. Furthermore, the figure implicitly indicates that the scorch time is inversely proportional to the curing temperature. This is easy to be understood that, for a given composite composition, e.g., CA1, at lower temperature more time is necessary to accumulate heat to achieve the required value of activation energy to initiate the vulcanization reaction for the respective compound.

Figure 12 shows that the optimum curing time (t_90_) is also inversely proportional to the composition of NR-g-St. This phenomenon, where t_90_ decreases as NR-g-St composition increases, agrees with previous discussion regarding M_H_. Since CA2 has the highest amount of NR-g-St, there were more NR backbones crosslinked with NF, bridged by the coupling agent. Therefore, it required the shortest time to achieve 90% of M_H_. Furthermore, the effect of the hybrid coupling agent to the vulcanization reaction was studied by calculating the difference between t_90_ and t_10_, in which t_10_ representing the time from the beginning of the curing reaction to the time where the torque reaches 10% above the lowest torque. This parameter can be used to qualitatively analyze the curing rate.

**Figure 13:**
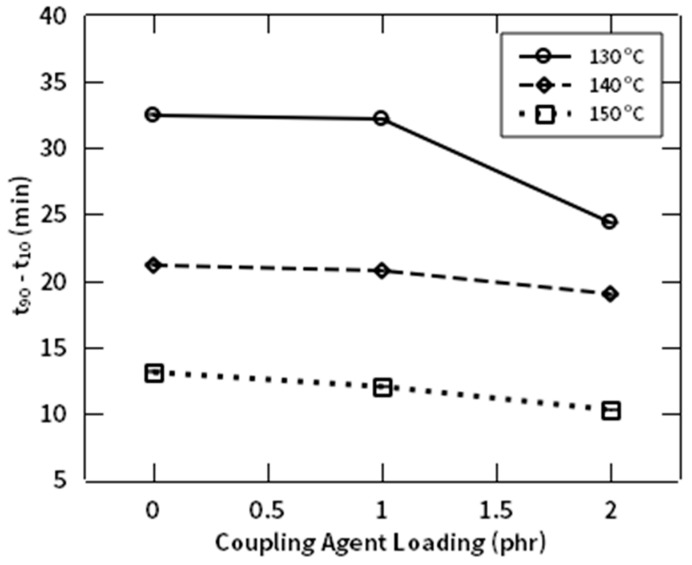
t_90_–t_10_ profile of NR compound at different composition of NR-g-St.

Figure 13 represents effect of the hybrid coupling agent to the curing rate of the composite compound (t_90_–t_10_). The curves indicate an acceleration of the vulcanization reaction through addition of NR-g-St into the composite compound. This tendency occurred at all temperatures of the rheological measurement, which indirectly confirm the analysis made for Figure 12.

The reduction of t_90_– t_10_ on composites that were incorporated with coupling agent was a result of two things. First, as mentioned before, adding NR-g-St to the rubber compound will lower the optimum curing time. Second, the presence of the coupling agents in the composites reduced the time required to reach the lowest torque (the induction point). These indicated that the addition of hybrid coupling agent was affecting the kinetical behavior of the crosslinking reaction. The next discussion is focused on the role of the hybrid coupling agent on accelerating the curing reaction through a kinetical modelling.

### 3.4. Modeling of Curing Kinetics

Obtaining quantitative measurement about the influence of NR-g-St on the curing kinetics of the rubber compound, the experiment was developed using a thermodynamic model. The first development step of the model was to investigate whether the curing reaction followed the first or higher reaction order. This step was performed by plotting the experimental data using the general equation for fist order chemical reaction, as shown in Equation (3) [41].

(3) ln(a−x)=−kt + ln a

where a, x, and k are the initial concentration, the reacted quantity of reactant at time t, and the first order rate constant, respectively. The term (a−x) in this case corresponds to the remaining uncured rubber, which was proportional to the difference between ${Μ}_{H}$ and the measured torque at time t (${Μ}_{t}$) as shown in Equation (4) [41].

(4) $$a-x={Μ}_{H}-{Μ}_{t}$$

**Figure 14:**
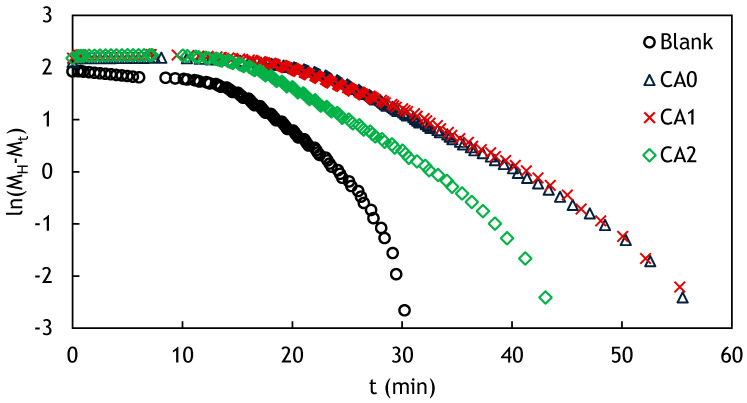
Kinetic plot of the composite compound added with NR-g-St at various compositions, which are measured at 150 °C.

Plots of ln(${Μ}_{H}-{Μ}_{t}$) against t show non-linier correlation, as shown in Figure 14. This implies that each vulcanization was not a first order reaction. Therefore, the Deng–Isayev approach, which accommodates higher order curing reaction, was used.

The Deng–Isayev approach is expressed by the following Equation (5) [42].

(5) $$\alpha =\frac{kt^{n}}{1+kt^{n}}$$

where α, k, and n are the state of cure, the curing rate constant and reaction order, respectively. State of cure refers to the extent of curing reaction of natural rubber matrix and can be determined by considering properties of rubber related with the crosslink density [43]. The state of cure can be measured by practical method through inserting parameters that were obtained from rheometer test, into Equation (6) [24],

(6) $$\alpha =\frac{M_{t}-M_{L}}{M_{H}-M_{L}}$$

**Figure 15:**
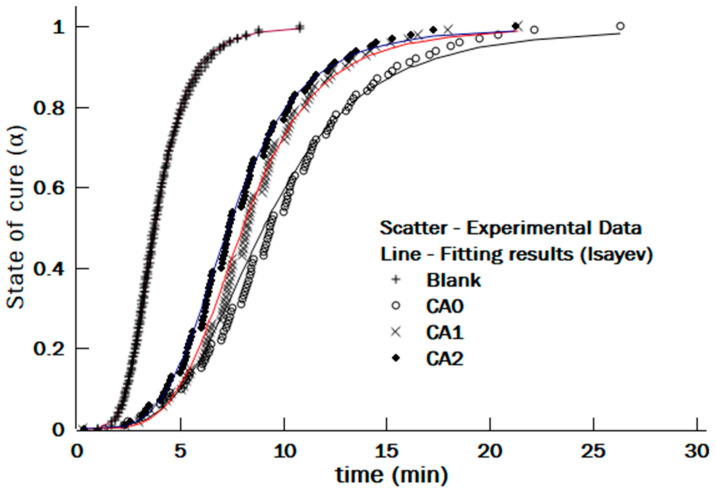
The fitting curves to calculate the state of cure for different NR-g-St loadings at 150 °C.

The value of $M_{L}$, as it marks the initial point of curing reaction, is used for measuring the state of cure. Hence the ($M_{t}-M_{L}$) in Equation (6) corresponds to the cured NR at time *t* and ($M_{H}-M_{L}$) represents the maximum curing state that can be achieved at a given curing temperature and NR compound composition. The results from Equation (6) were plotted against *t* and then fitted using Equation (5). Fitting level of the predicted state of cure was measured using Ordinary Least Square (OLS) as shown in Equation (7) [44],

(7) $$OLS=\sum {\left(\alpha_{\exp}\left(t\right)-\alpha_{\mathrm{model}}\left(t\right)\right)}^{2}$$

where $\alpha_{\exp}\;$ is the experimental value and $\alpha_{\mathrm{model}}$ is the prediction of the model. The fitting curves for different NR-g-St loadings at 150 °C are shown in Figure 15.

Figure 15 implies that the experimental data were well fitted using Equation (5). The degree of fitting between the experimental and the calculated data were then verified by the coefficient of determination, R^2^, as presented in Table 2. All values of the R^2^, which were above 95% (0.950), concluded that the Deng–Isayev model is eligible as a model for the curing reactions in the composite NR compounds. In addition, the average OLS values for Blank, CA0, CA1, and CA2 were 0.052, 0.047, 0.057, and 0.036, respectively.

As shown in Table 2, comparison of the activation energies resulted from the kinetic modelling indicated that addition of the hybrid coupling agent into the NR composite compounds had an effect to lower the activation energy for the curing reactions in the NR composites. This result was in line to what has been discussed in Section 3.3, where time for the curing reactions in the rubber composite with the hybrid coupling agent was shorter than the one without the hybrid coupling agent. This phenomenon may be caused by non-polar and polar covalent bonds in NR-g-St promoting the formation of intermolecular bonding between the polar part and the sorghum fiber. This interaction may ease the NR crosslinking through decreasing the activation energy for the curing reactions. As shown in Table 2, there was no linear relationship between the hybrid coupling agent composition and the activation energy for the curing reaction. This finding may have been caused by the crosslinking reactions occurring in NR composite compounds, which have some reaction with complex mechanisms, as discussed in Figure 1. This phenomenon is in line as reported by Wu et al. [25].

## 4. Conclusions

The incorporation of starch-rubber coupling agent to NR/NF composites successfully improved the compatibility between the NF and NR matrix. The FTIR spectrum showed peaks in wavenumber range of 2830–2970 cm^−1^, which corresponded to –CH_2_ and –CH_3_ bonds, are obviously found for crosslinked NR composite. It was presumably caused by the intermolecular interaction during vulcanization as the result of modifying the structure of NR backbone. The FE-SEM images exhibited that the presence of hybrid materials in NR/NF compounds improved the fibers and matrix compatibility indicated by no noticeable gaps among surfaces of the fiber and the matrix.

The addition of the starch-rubber coupling agent gives an effect to ease curing reaction through decreasing the curing activation energy. The rheological measurements in this study implied that the novel coupling agent addition to NR/NF compound was able to shorten the curing rate of the compound (t_90_–t_10_) from 33 min (CA0) to 10 min, which was achieved at highest composition of coupling agent and temperature, viz. 2 phr and 150 °C, respectively. The modelling study indicated that the positive effects of the coupling agent addition to the curing behavior may be caused by decreasing the curing activation energy until 4.71 × 10^5^ kJ/mol.

Results obtained from this study promises to strengthen the composite-based rubber products such as tires in enhancing the mechanical and the physical properties. The natural fibers also have excellent acoustic properties in absorbing sounds for example noise generated from friction between tires and road. Considering those properties, the further study would be focused on effect of the novel coupling agent in the sorghum fiber-based rubber composites, to the properties, and then their applications.

## Figures and Tables

**Table 1:** Compositions of prepared samples (in phr).

	Natural Rubbers (NR)	Natural Fiber (NF)	NR-g-St	ZnO	Stearic Acid	CBS	Sulfur
Blank	100	-	-	6	0.5	0.7	2.5
CA0	100	20	-	6	0.5	0.7	2.5
CA1	100	20	1	6	0.5	0.7	2.5
CA2	100	20	2	6	0.5	0.7	2.5

**Table 2:** Kinetic parameters for the curing reactions in the composite NR compound in various NR-g-St loadings and measurement temperatures.

Sample	T(°C)	n	$k\;\times \;10^{-16}(s^{-1})$	$R^{2}$	$E_{0}\;\times \;10^{5}\;(\mathrm{kJ}/\mathrm{mol})$
Blank	130	5.476	1.1	0.981	7.34
	140	5.121	313.99	0.983	
	150	4.862	34,281.51	0.994	
CA0	130	5.143	0.33	0.994	10.09
	140	4.709	125.13	0.991	
	150	3.748	559,255.37	0.973	
CA1	130	4.677	12.48	0.994	4.71
	140	4.751	101.73	0.975	
	150	4.474	9821.82	0.977	
CA2	130	5.239	0.59	0.990	8.00
	140	4.642	250.73	0.984	
	150	4.284	46,740.28	0.989	
